# Effect and Comparison of Luteolin and Its Derivative Sodium Luteolin-4′-sulfonate on Adipogenic Differentiation of Human Bone Marrow-Derived Mesenchymal Stem Cells through AMPK-Mediated PPAR*γ* Signaling

**DOI:** 10.1155/2020/8894910

**Published:** 2020-10-29

**Authors:** Jung Hwan Oh, Fatih Karadeniz, Jung Im Lee, Youngwan Seo, Mi-Soon Jang, Chang-Suk Kong

**Affiliations:** ^1^Marine Biotechnology Center for Pharmaceuticals and Foods, College of Medical and Life Sciences, Silla University, Busan 46958, Republic of Korea; ^2^Division of Marine Bioscience, Korea Maritime and Ocean University, Busan 49112, Republic of Korea; ^3^Food Safety and Processing Research Division, National Institute of Fisheries Science, Busan 46083, Republic of Korea; ^4^Department of Food and Nutrition, College of Medical and Life Sciences, Silla University, Busan 46958, Republic of Korea

## Abstract

Luteolin is a common phytochemical from the flavonoid family with a flavone structure. Studies reported several bioactivities for luteolin and similar flavones. Attenuating the increased adipogenesis of bone marrow cells (hBM-MSCs) has been regarded as a therapeutic target against osteoporotic bone disorders. In the present study, the potential roles of luteolin and its sulfonic acid derivative luteolin-OSO_3_Na in regulating adipogenic differentiation of hBM-MSCs were investigated. Adipo-induced cells were treated with or without compounds, and their effect on adipogenesis was evaluated by adipogenic marker levels such as lipid accumulation and PPAR*γ* pathway activation. Luteolin hindered the adipogenic lipid accumulation in adipo-induced hBM-MSCs. Immunoblotting and reverse transcription-polymerase chain reaction analysis results indicated that luteolin downregulated PPAR*γ* and downstream factors of C/EBP*α* and SREBP1c expression which resulted in inhibition of adipogenesis. Luteolin-OSO_3_Na showed similar effects; however, it was significantly less effective compared to luteolin. Investigating p38, JNK, and ERK MAPKs and AMPK activation indicated that luteolin suppressed the MAPK phosphorylation while stimulating AMPK phosphorylation. On the other hand, luteolin-OSO_3_Na was not able to notably affect the MAPK and AMPK activation. In conclusion, this study suggested that luteolin inhibited adipogenic differentiation of hBM-MSCs via upregulating AMPK activation. Replacing its 4′-hydroxyl group with sulfonic acid sodium salt diminished its antiadipogenic effect indicating its role in regulating AMPK activation. The general significance is that luteolin is a common phytochemical with various health-beneficial effects. The current study suggested that luteolin may serve as a lead compound for developing antiosteoporotic substances with antiadipogenic properties.

## 1. Introduction

Obesity is a worldwide health problem, and many diseases such as cardiovascular disease, liver and kidney diseases, diabetes, and cancer are considered to be linked with the prior cases of excess body fat accumulation [1]. Obesity development occurs through high rates of proliferation and differentiation of white adipocytes, which result in expanding white adipose tissue. Negating the effects of obesity while elucidating the underlying mechanism and preventing the formation of adipose tissue has been of great interest in the pharmaceutical field [2]. In addition to adipocyte differentiation in adipose tissue itself, obesity-linked complications are known to meditate the other adipogenesis-related disorders including osteoporosis, informally labeled as obesity of the bone [3]. Activated adipocyte differentiation mechanisms whether due to obesity or not result in an elevated number of adipocytes in bone marrow compared to osteoblasts and osteocytes. It was also showed that mesenchymal stromal cells of other organs such as bone marrow can play roles in adipose tissue homeostasis through adipogenic differentiation [4]. Cultured human bone marrow-derived mesenchymal stromal cells (hBM-MSCs), therefore, are reliable and well-known in vitro models for studying human adipogenesis mechanisms and ways to hinder it.

Most of the natural products with pharmaceutical potential against metabolic syndrome-linked complications are of plant origin. These substances including but not limited to flavonoids, coumarins, and terpenes have been shown to possess therapeutic properties for the symptoms of obesity, diabetes, cardiovascular diseases, and osteoporosis [5, 6]. Different types of flavonoids, a very common polyphenol secondary metabolite found in plants and fungus, are included in several commercial preparations with medical, nutritional, and cosmeceutical uses [7]. Studies have reported that some of these flavonoids exert antiadipogenic properties via different mechanisms of action depending on the chemical structure of the compound [8, 9]. Luteolin is a known flavonoid with reported health-beneficial effects such as anti-inflammatory, anticancer, and antiallergy [10]. Studies have shown that luteolin inhibited the adipogenic differentiation of mouse preadipocyte cells [11] as well as inducing browning and thermogenesis in mice adipose tissue [12]. Also, several derivatives of luteolin, such as orientin which is an 8-C-glucoside derivative, were reported to have biological activities comparable to that of luteolin [13]. However, to the best of our knowledge, inhibition of mesenchymal stromal cell adipogenesis, its mechanism, and structure-activity relationship have yet to be studied. Therefore, the current study was aimed to investigate the possible antiadipogenic properties and underlying mechanisms of luteolin and its sulfate derivative, luteolin-4′-sulfonate, using adipogenesis-induced human bone marrow-derived mesenchymal stem cells (hBM-MSCs).

## 2. Materials and Methods

### 2.1. Luteolin and Luteolin-OSO^3^Na

**Figure 1:**
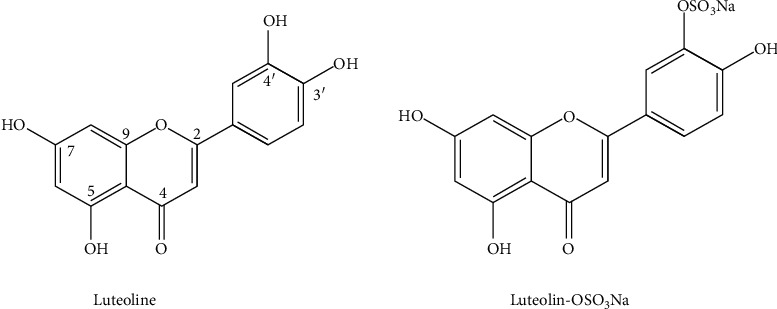
Chemical structures of luteolin and luteolin-OSO_3_Na [15].

Luteolin and luteolin-OSO_3_Na were isolated from *Zostera asiatica*, and their chemical structures were elucidated as previously reported [14] (Figure 1).

### 2.2. Cell Culture and Adipogenic Differentiation

Bone marrow-derived human mesenchymal stem cells (hBM-MSCs) were procured from PromoCell (C-12974, Heidelberg, Germany). Cells were seeded in 6-well plates (1 × 10^6^ cells/well) and cultured using Mesenchymal Stem Cell Growth Medium (C-28009, PromoCell). Incubation of the plates was carried out in an environment with 37°C temperature and 5% CO_2_ atmosphere. For adipogenic differentiation of hBM-MSCs, cells were grown to confluence prior to swapping cell culture medium with Mesenchymal Stem Cell Adipogenic Differentiation Medium 2 (C-28016, PromoCell). Following the introduction of differentiation, medium cells were incubated for 10 days (unless otherwise noted), and the medium was changed every third day without disturbing the cell monolayer. Luteolin and luteolin-OSO_3_Na were supplied along with initial differentiation inducement and were not present in consequent media changes.

### 2.3. Cell Viability Assay

The effect of samples on the viability of hBM-MSC cells was investigated using common MTT assay procedures. Cells were seeded in 96-well plates (1 × 10^3^ cell/well) and incubated for 24 h which was followed by the sample treatment. The viability of the treated and untreated cells was quantified after 24 h incubation. Briefly, wells were aspirated after 24 h treatment and were supplied with 100 *μ*L of MTT reagent (1 mg/mL). Plates were then kept under dark for 4 h at room temperature. Viable cell-dependent formation of formazan salts was quantified by the addition of 100 *μ*L DMSO to each well and measurement of the absorbance value at 540 nm with a microplate reader (Multiskan GO, Tecan Austria GmbH, Grodig, Austria).

### 2.4. Oil Red O Staining

Display of the accumulated intracellular lipid droplets by adipocytes was carried out with common Oil Red O staining protocols. Briefly, cells were cultured in 6-well plates and differentiated into adipocytes as previously described. Following 10 days of differentiation, wells were aspirated and washed with PBS followed by cell fixation via addition of 10% fresh formaldehyde (in PBS, v/v). Fixation was continued for 1 h at room temperature. Staining of the lipid droplets was performed by the addition of 1 ml Oil Red O solution (prepared in 6 parts of isopropanol and 4 parts of water) into aspired and washed (with PBS) wells. After 1 h, Oil Red O staining solution was removed, and wells were air-dried. Stained lipid droplets were visualized under an optical microscope (Olympus, Tokyo, Japan). The stain from the lipid droplets was eluted by the presence of 100% isopropyl alcohol in wells. Quantification was carried out by colorimetry, measuring the absorbance of the wells (containing retained dye and 100% isopropyl alcohol) at 500 nm using a microplate reader (Multiskan GO).

### 2.5. Reverse Transcription-Polymerase Chain Reaction Assay

Total RNA was extracted using the AccuPrep Universal RNA Extraction Kit (Bioneer Corp., Daejeon, Republic of Korea) from cells at day 10 of differentiation, and the cDNA synthesis from total RNA (2 *μ*g) was carried out with Cell Script cDNA master mix (CellSafe, Gyeonggi-do, Republic of Korea) in a T100 Thermal Cycler (Bio-Rad Laboratories, Inc., Hercules, CA, USA). The following temperature protocol was used for reverse transcription: 42°C for 60 min and 72°C for 5 min. Subsequently, PCR was performed using the following primers: forward 5′-TTT-TCA-AGG-GTG-CCA-GTT-TC-3′ and reverse 5′-AAT-CCT-TGG-CCC-TCT-GAG-AT-3′ for PPAR*γ*; forward 5′-TGT-TGG-CAT-CCT-GCT-ATC-TG-3′ and reverse 5′-AGG-GAA-AGC-TTT-GGG-GTC-TA-3′ for SREBP1c; forward 5′-TTA-CAA-CAG-GCC-AGG-TTT-CC-3′ and reverse 5′-GGC-TGG-CGA-CAT-ACA-GTA-CA-3′ for C/EBP*α*; and forward 5′-CCA-CAG-CTG-AGA-GGG-AAA-TC-3′ and reverse 5′-AAG-GAA-GGC-TGG-AAA-AGA-GC-3′ for *β*-actin. The following thermocycling conditions were used for PCR: 30 cycles of 95°C for 45 sec, 60°C for 1 min, and 72°C for 45 sec. The final PCR products were separated by electrophoresis for 30 min at 100 V on a 1.5% agarose gel. Following staining with 1 mg/ml ethidium bromide, gels were imaged under a UV light from CAS-400SM Davinch-Chemi Imager™ (Davinch-K, Seoul, Korea). Bands on gels were quantified by densitometric analysis with MultiGauge software (v3.0; Fujifilm, Tokyo, Japan).

### 2.6. Immunoblotting

Detection of protein expression in cells was carried out with standard Western blotting techniques. Briefly, hBM-MSCs at day 10 differentiation were lysed by vigorous pipetting with 1 ml RIPA buffer (Sigma-Aldrich; Merck KGaA) at 4°C to obtain whole-cell lysates. Total protein was quantified using a BCA protein assay (Thermo Fisher Scientific, Inc.) according to the manufacturer's protocol. Proteins (20 *μ*g) were separated via 12% SDS-PAGE at 100 V and transferred to PVDF membranes (Cytiva Life Sciences, Marlborough, MA, USA). Membrane background was blocked with 5% skimmed milk, and the membranes were washed with 1X TBST (0.1% Tween-20) prior to incubation with primary antibodies. Primary antibody incubation was carried out in a buffer containing 1X TBST with 5% BSA (Sigma-Aldrich; Merck KGaA) overnight at 4°C. The following primary antibodies were used: PPAR*γ* (#2443; Cell Signaling Technology, Danvers, MA, USA), CCAAT/enhancer-binding protein (C/EBP)*α* (#2295; Cell Signaling Technology), sterol regulatory element-binding protein-1c (SREBP1c) (ab3259; Abcam, Cambridge, England, UK), p38 (#8690; Cell Signaling Technology), phospho(p)-p38 (#4511; Cell Signaling Technology), JNK (LF-PA0047; Thermo Fisher Scientific), p-JNK (sc-293136; Santa Cruz Biotechnology, Santa Cruz, CA, USA), ERK (#4695; Cell Signaling Technology), p-ERK (#4370; Cell Signaling Technology), AMPK (#2603; Cell Signaling Technology), p-AMPK (#2531; Cell Signaling Technology), and *β*-actin (sc-47778; Santa Cruz Biotechnology). Subsequently, the membranes were incubated with HRP-conjugated secondary antibodies for 1 h at room temperature. The following source-specific secondary antibodies were used: anti-mouse (cat. no. #7076; Cell Signaling Technology, Inc.), anti-rabbit (cat. no. #7074; Cell Signaling Technology, Inc.), and anti-goat (cat. no. sc-2354; Santa Cruz Biotechnology, Inc.). Protein bands were stained with an ECL Western blot detection kit (Cytiva Life Sciences), and the stained bands were observed with CAS-400SM Davinch-Chemi imager (Davinch-K). Bands were quantified via densitometric analysis using MultiGauge software (v3.0; Fujifilm, Tokyo, Japan).

### 2.7. Immunofluorescence Staining

Detection of perilipin-1 and PPAR*γ* expression in adipo-induced hBM-MSCs was observed by immunofluorescence staining. Cells were grown and induced to differentiate on glass coverslips . At day 10 of differentiation, cells were fixed and stained with anti-perilipin-1 (ab3526; Abcam) and anti-PPAR*γ* antibody (ab9256; Abcam) conjugated with Alexa Fluor 488 (A-11008; Invitrogen) and ProLong Gold Antifade Reagent with DAPI (#8961; Cell Signaling Technology) to highlight the nuclei. Fixation and staining of the cells were carried out using immunofluorescence application solution kit (#12727; Cell Signaling Technology), according to the manufacturer's instructions.

### 2.8. Flow Cytometry

The MAPK (ERK1/2) activation levels were investigated by employing flow cytometry. hBM-MSCs were seeded in 6-well plates (1 × 10^6^ cell/well) and induced to differentiate following confluence. At day 10 of differentiation, levels of ERK1/2 phosphorylation were measured with the MUSE^TM^ MAPK Activation Dual Detection Kit (MCH200104; Merck KGaA, Darmstadt, Germany) using MUSE^TM^ Cell Analyzer and software (Muse Cell Soft V1.4.0.0, Merck KGaA, Darmstadt, Germany) according to the manufacturer's instructions.

### 2.9. Statistical Analysis

Data were given as mean of three experiments ± SD unless otherwise noted. Groups in the same data series were subjected to one-way analysis of variance (ANOVA) with post hoc Duncan's test for the determination of statistically significant difference which was defined at *p* < 0.05 level (SAS v9.1, SAS Institute, Cary, NC, USA).

## 3. Results and Discussion

### 3.1. Inhibition of Adipogenesis and PPAR*γ* Signaling

**Figure 2:**
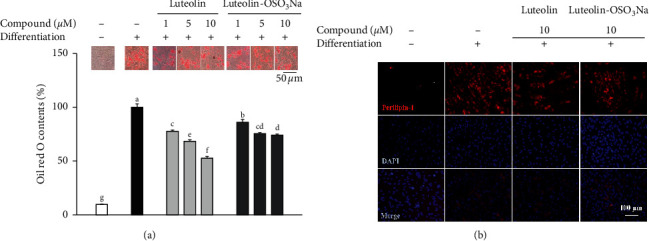
Effect of luteolin and luteolin-OSO_3_Na on the lipid accumulation of adipo-induced hBM-MSCs. (a) Cells were seeded in 6-well plates and induced to differentiate with adipocyte differentiation medium in the absence or presence of compounds (1, 5, and 10 *μ*M). Following 10 days of incubation, intracellular lipid droplets of mature adipocytes were stained with Oil Red O. Lipid accumulation levels were calculated by the colorimetric quantification of the dye removed from the wells and given as percentage of adipo-induced untreated control group. Values are means ± SD (*n* = 3). Different letters (A–E) indicate statistically significant difference (*p* < 0.05) by Duncan's multiple range test. (b) Fluorescence micrographs of the adipo-induced hBM-MSCs at differentiation day 10, stained with FITC-conjugated anti-perilipin-1 antibody (red) and DAPI (blue) to highlight the nuclei. Scale bar, 100 *μ*m [15].

Adipo-induced hBM-MSCs showed intracellular lipid droplets detectable under a light microscope following Oil Red O staining at day 10 of differentiation (Figure 2(a)). In addition to intracellular fat accumulation as droplets, cellular morphology was also changed towards being more spherical. Treatment of differentiating hBM-MSCs with luteolin and luteolin-OSO_3_Na decreased the accumulated lipid droplets in a dose-dependent manner. At the concentration of 10 *μ*M, luteolin-treated cells had 47.24% less intracellular lipid content compared to untreated control adipocytes. This decrease was observed to be 25.98% in terms of luteolin-OSO_3_Na at the same concentration. Results indicated that luteolin treatment hindered the adipogenic characteristics of differentiating hBM-MSCs hinting at inhibition of adipocyte maturation. However, addition of SO_3_Na to hydroxyl chain of the luteolin phenyl ring (Figure 1) lowered the efficiency of luteolin inhibition on lipid accumulation. The inhibitory effect of samples on lipid accumulation was further assessed by the immunofluorescence staining of perilipin-1 in differentiated hBM-MSCs. Perilipin-1 is a coating protein for the lipid droplets and hence is a marker for the successful formation of lipid droplets in adipo-induced hBM-MSCs [16]. Staining results showed that adipo-induced hBM-MSCs showed the increased amounts of lipid droplets compared to the nondifferentiated untreated control group (Figure 2(b)). Similar to Oil Red O staining results, treatment with luteolin and luteolin-OSO_3_Na decreased the perilipin-1 staining, indicating lesser lipid droplets.

**Figure 3:**
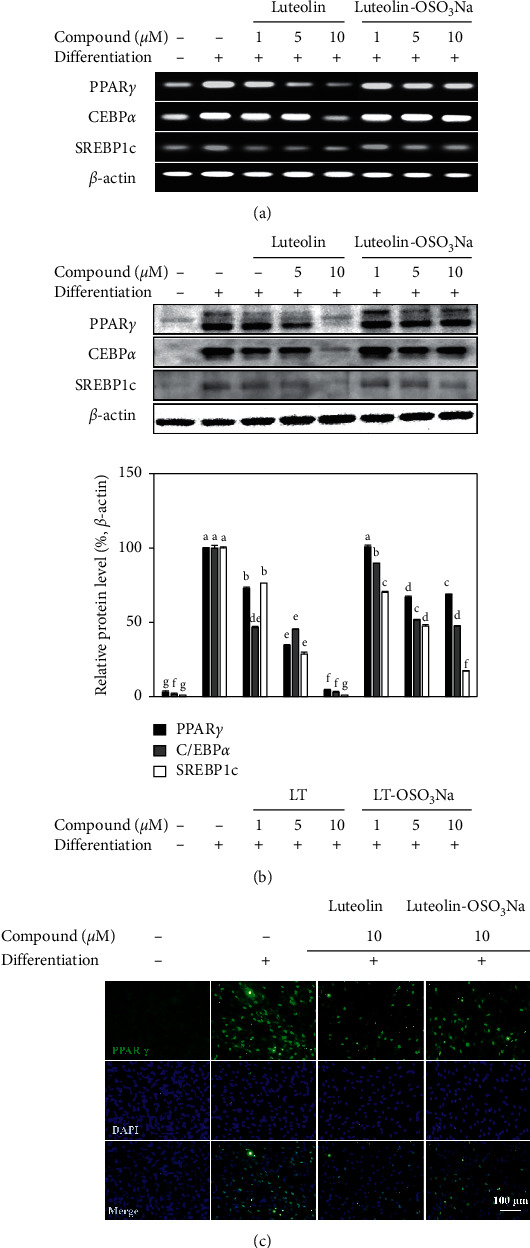
Effect of luteolin and luteolin-OSO_3_Na on the expression levels of key adipogenic differentiation markers, PPAR*γ*, C/EBP*α*, and SREBP1c in adipo-induced hBM-MSCs. Cells were seeded in 6-well plates and induced with adipocyte differentiation medium in the absence or presence of compounds (1, 5, and 10 *μ*M). Following 10 days of incubation, cell lysates were used for the detection of PPAR*γ*, C/EBP*α*, and SREBP1c mRNA (a) and protein (b) levels employing RT-PCR and western blotting, respectively. *β*-Actin was used as internal loading control. Values are means ± SD (*n* = 3). Different letters (A–G) indicate statistically significant difference (*p* < 0.05) by Duncan's multiple range test. (c) Fluorescence micrographs of the adipo-induced hBM-MSCs at differentiation day 10, stained with FITC-conjugated anti-PPAR*γ* antibody (green) and DAPI (blue) to highlight the nuclei. Scale bar, 100 *μ*m [15].

To determine whether a decrease in lipid accumulation by luteolin was due to inhibition of adipogenic differentiation, PPAR*γ* signaling was examined. Following the adipogenic stimulation of hBM-MSCs, cells were treated with luteolin and luteolin-OSO_3_Na. The mRNA and protein expressions of PPAR*γ*, C/EBP*α*, and SREBP1c were then analyzed by RT-PCR and western blotting, respectively. It had been reported that PPAR*γ*, C/EBP*α*, and SREBP1c were crucial transcription factors for adipogenesis and had important roles in obesity and diabetes [17]. Increased expression of these transcription factors is essential at the early stages of adipogenesis, and activation of PPAR*γ* subsequently stimulates the expression of C/EBP*α* and SREBP1c [18]. Treatment of adipo-induced hBM-MSCs with both luteolin and luteolin-OSO_3_Na reduced the mRNA expression of PPAR*γ*, C/EBP*α*, and SREBP1c (Figure 3(a)). This outcome was also confirmed by western blotting (Figure 3(b)). Similar to lipid accumulation inhibition, the suppression effect of luteolin-OSO_3_Na was significantly lower than that of luteolin, suggesting a similar mechanism. Studies on different flavonoids with similar chemical structures had reported adipogenesis inhibitory effects in murine preadipocytes [8, 9]. Among them, quercetin which has a similar structure to luteolin was shown to inhibit lipid accumulation via activation of lipid catabolism as well as direct inhibition of PPAR*γ*-mediated adipogenesis [19, 20]. PPAR*γ* inhibitory effect of the samples was further confirmed by the immunofluorescence staining of PPAR*γ* in adipo-induced hBM-MSCs. Both luteolin and luteolin-OSO_3_Na showed decreased PPAR*γ* levels in hBM-MSC adipocytes compared to the untreated adipo-induced control group (Figure 3(c)). However, Park et al. [21] had reported that luteolin did not affect lipid catabolism in 3T3-L1 preadipocytes unlike quercetin [20] and suggested different action mechanisms such as AMPK.

### 3.2. Inhibition of MAPK Activation

**Figure 4:**
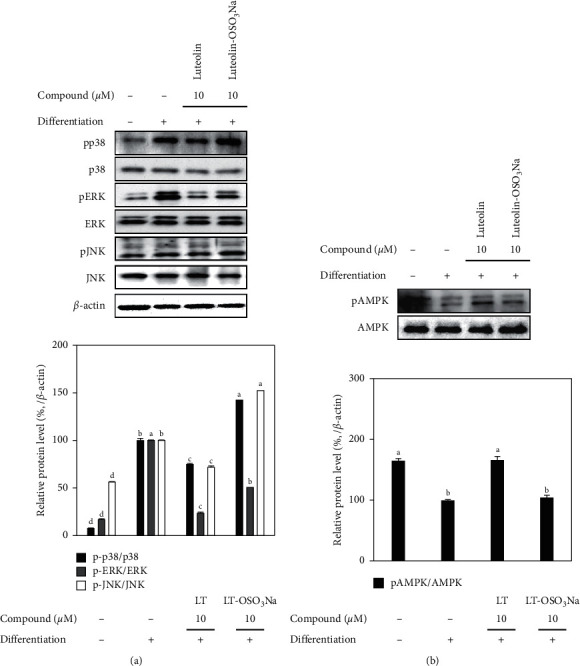
Effect of luteolin and luteolin-OSO_3_Na on the protein levels of MAPK and AMPK proteins in adipo-induced hBM-MSCs. Cells were seeded in 6-well plates and induced with adipocyte differentiation medium in the absence or presence of compounds (10 *μ*M). Following 10 days of incubation, cell lysates were used for the detection of total and phosphorylated p protein levels of p38, ERK, JNK, and AMPK using western blotting. *β*-Actin was used as internal loading control. Values are means ± SD (*n* = 3). Different letters (A–D) indicate statistically significant difference (*p* < 0.05) by Duncan's multiple range test [15].

**Figure 5:**
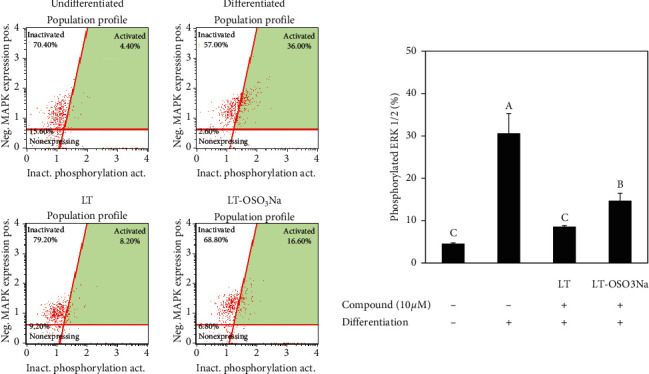
Effect of luteolin and luteolin-OSO_3_Na on the activation of MAPK (ERK1/2) in adipo-induced hBM-MSCs at day 10 of differentiation analyzed by FACS flow cytometry. Plots give the percentage of cells with phosphorylated ERK1/2 in the total population. Values are means ± SD (*n* = 3). Different letters (A–C) indicate statistically significant difference *p* < 0.05) by Duncan's multiple range test [15].

In order to provide insights towards possible action of mechanisms underlying the antiadipogenic properties of luteolin and the relation between its chemical structure and bioactivity, the effect of luteolin and luteolin-OSO_3_Na on MAPK activation was investigated. Reports had shown that MAPK activation promoted or inhibited the adipogenic differentiation depending on the stimulatory signal [22]. Current results showed that adipogenic stimulation of hBM-MSCs resulted in increased phosphorylation of p38, ERK, and JNK MAPKs (Figure 4(a)). Treatment with 10 *μ*M luteolin downregulated the activation of MAPKs. Luteolin-OSO_3_Na was also shown to suppress ERK activation albeit not as strong as luteolin while being unable to affect p38 and JNK activation. Results suggested that luteolin inhibited the adipogenesis in hBM-MSCs via downregulation of MAPKs. MAPK-linked adipogenesis inducement occurs along with downregulated AMPK activation [23]. In this context, the effect of luteolin and luteolin-OSO_3_Na on AMPK activation was also analyzed by western blotting. Adipo-induced hBM-MSCs expressed decreased levels of phosphorylation of AMPK which was reverted by 10 *μ*M luteolin treatment (Figure 4(b)). On the other hand, luteolin-OSO_3_Na treatment slightly increased AMPK phosphorylation, however not comparable to luteolin. These results were parallel to MAPK activation as reports had shown that activation of p38, ERK, and JNK MAPKs during adipogenesis was in the opposite manner with AMPK activation [15, 23]. Effect of samples on MAPK activation was further analyzed by flow cytometry assessing the expression of phosphorylated MAPK (ERK1/2) in adipo-induced hBM-MSCs. The role of MAPK/ERK signaling cascade in obesity was also suggested by Hirosumi et al. [24] where the deletion of JNK1 gene of MAPK/ERK signaling resulted in significant improvements observed as decreased adiposity and improved insulin sensitivity *in vivo*. Results showed that luteolin treatment (10 *μ*M) decreased the activated ERK1/2 levels to 8.53% from 30.60% of untreated hBM-MSC adipocytes (Figure 5). At the same conditions, the luteolin-OSO_3_Na-treated group showed 14.67% activated ERK1/2.

Therefore, present results suggested that luteolin treatment inhibited the adipogenesis of hBM-MSCs via downregulation of PPAR*γ* signaling through activation of AMPK. Luteolin-OSO_3_Na, however, showed notably decreased efficiency in inhibiting adipogenesis in hBM-MSCs and was unable to significantly alter MAPK and AMPK activation, indicating the involvement of hydroxy chain at 4′ of phenyl ring. Further *in vivo* studies are needed to elucidate and confirm the mechanism of luteolin and luteolin-OSO_3_Na antiadipogenic activity. The comparison with luteolin-OSO_3_Na might lack the necessary data to conclude on the role of dihydroxyphenyl ring of luteolin in its AMPK-mediated activity. Therefore, using different dihydroxyphenyl ring derivatives will provide insights towards its structure-activity relationship. Nevertheless, current results might serve as a base for further studies to develop luteolin-based nutraceutical agents against obesity.

## 4. Conclusion

The current study showed that luteolin could inhibit adipogenic differentiation of hBM-MSCs, indicating a possible action against osteoporotic obesity of the bone. Results suggested that luteolin exerted its effect via downregulation of PPAR*γ* cascade through stimulation of AMPK activation. Although inhibition of lipid accumulation and PPAR*γ* signaling downregulation were observed with luteolin-OSO_3_Na treatment, it was not as effective as luteolin. In conclusion, luteolin was reported to be a potential antiadipogenic compound. It was also suggested that its dihydroxyphenyl ring plays important roles in regulating AMPK-mediated adipogenesis inhibition.

## Data Availability

All data used to support the findings of this study are available from the corresponding author upon reasonable request.
